# Evaluating Population Density as a Parameter for Optimizing COVID-19 Testing: Statistical Analysis

**DOI:** 10.2196/22195

**Published:** 2021-02-03

**Authors:** Karim I Budhwani, Henna Budhwani, Ben Podbielski

**Affiliations:** 1 CerFlux Inc Birmingham, AL United States; 2 University of Alabama at Birmingham Birmingham, AL United States; 3 Protective Life Corp Birmingham, AL United States

**Keywords:** infectious diseases, testing, per capita, population density, policy, coronavirus, SARS-CoV-2, COVID-19

## Abstract

**Background:**

SARS-CoV-2 transmission risk generally increases with the proximity of those shedding the virus to those susceptible to infection. Thus, this risk is a function of both the number of people and the area they occupy. However, the latter continues to evade the COVID-19 testing policy.

**Objective:**

The aim of this study is to analyze per capita COVID-19 testing data reported for Alabama to evaluate whether testing realignment along population density, rather than density agnostic per capita, would be more effective.

**Methods:**

Descriptive statistical analyses were performed for population, density, COVID-19 tests administered, and positive cases for all 67 Alabama counties.

**Results:**

Tests reported per capita appeared to suggest widespread statewide testing. However, there was little correlation (*r*=0.28, *P*=.02) between tests per capita and the number of cases. In terms of population density, new cases were higher in areas with a higher population density, despite relatively lower test rates as a function of density.

**Conclusions:**

Increased testing in areas with lower population density has the potential to induce a false sense of security even as cases continue to rise sharply overall.

## Introduction

COVID-19 testing is typically measured per capita; tests and cases are reported per million globally while local authorities report counts per 100,000 people [1-3]. This approach is simple and generally well accepted both in economic spheres and in health care research. However, this simplicity may shroud an underlying fallacy in applying per capita models to test the transmission characteristics of SARS-CoV-2. The transmission risk profile for 20 people in an elevator is substantially different from that of 20 people spread across a football field; this was the fundamental premise for social distancing and lockdowns to “flatten the curve.” Moreover, population density can impede [4] implementation of protective distancing measures. Population density has also been implicated [5] in COVID-19 mortality. In this two-part study, we analyze per capita COVID-19 testing data reported for Alabama to evaluate whether testing realignment along population density, rather than density agnostic per capita, would be more effective, as Alabama is one of several states currently experiencing notable increases in new cases.

## Methods

Population characteristics and population density for all 67 Alabama counties were obtained from the 2018 American Community Survey (US Census Bureau). The number of tests administered and positive cases of COVID-19 are updated daily by the Alabama Department of Public Health. These data were obtained on May 18, 2020, for initial assessment and again on June 15, 2020, for prospective analysis. Descriptive statistical analyses were performed to calculate the total number of tests per 100,000 people using the county population as the denominator, and subsequently dividing this by county population density, density squared, and square root of density as illustrative proxies [6,7] of more complex population density test rate models. All study data were publicly available, thereby obviating institutional review board approval.

## Results

**Figure 1:**
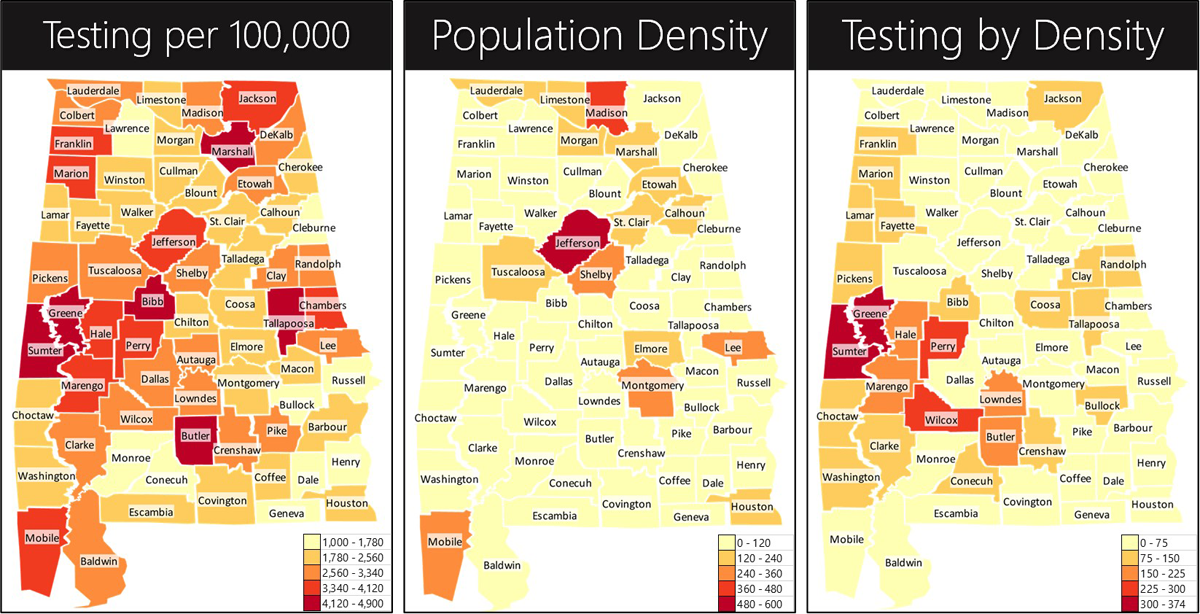
Per capita and population density heatmaps for COVID-19 tests between April 1 and May 18, 2020. (A) Heatmap of tests per 100,000. (B) Population density heatmap distinguishing sparsely populated areas from those that are densely populated. (C) Overlaying the two shows current testing by population density. Without a population density–driven testing approach, the risk of deriving a false sense of security is greater.

The first heatmap presented in Figure 1 appears to indicate widespread testing per 100,000 people [8] by county. However, this heatmap does not distinguish sparsely populated areas that could inherently provide spatial distancing from those that are densely populated (Figure 1B) [9]. Overlaying the two (Figure 1C) provides a sense of magnitude by which we may be overtesting in areas with a natural spatial defense against transmission while severely undertesting in areas with an elevated risk of transmission.

**Figure 2:**
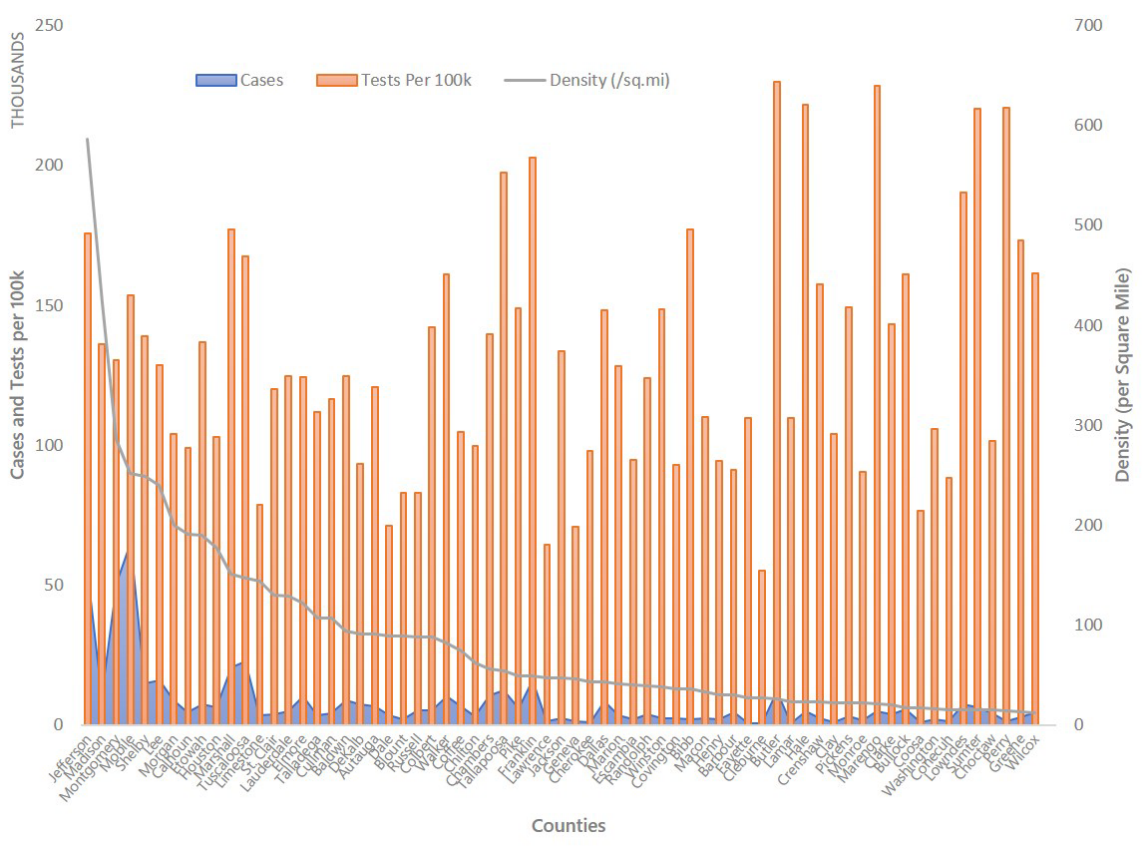
COVID-19 testing during the phased reopening of the Alabama economy from May 18 to June 15, 2020. Tests reported per 100,000 during this period also appeared to indicate widespread statewide testing. However, there was little correlation (*r*=0.28, *P*=.02) between tests per capita and the number of cases. In terms of population density, new cases were higher in areas with higher population density, despite relatively lower test rates as a function of density. This suggests that a population density–driven testing strategy would not only allow for more effective allocation but could also reduce the risk of understating cases in areas with high population density.

In the second part of the study, conducted during the phased economic re-engagement, data were collected to prospectively analyze the distribution of tests and cases vis-à-vis population density. Tests reported per 100,000 during this period, once again, appeared to indicate widespread statewide testing. However, there was little correlation (*r*=0.28, *P*=.02) between tests per capita and the number of cases. As anticipated [10], new cases were disproportionately more prevalent in densely populated areas (Figure 2), despite relatively fewer tests per population density, suggesting that cases in these areas may be understated.

## Discussion

The current standard of population density agnostic per capita reporting could induce a sense of false security while simultaneously accelerating infection in economic nerve centers. The contrast among the heatmaps, as well as subsequent prospective analysis of tests and cases, unveil the scale of testing disparity. A robust testing strategy would presumably figure prominently in the calculus for any phased reopening of economies and associated near-term paths to societal normalcy and economic recovery. Consequently, disparities in testing induced by a density agnostic testing approach could undermine balancing measures aimed at saving lives and livelihoods, thereby leading to a prolonged recession, or dare we say, a depression [11,12].

Although we use Alabama for illustration, most states report statistics in this manner, making our processes replicable in other states. This said, limitations of our approach should be considered when extending findings. Namely, population density–driven testing has not be extensively evaluated for feasibility and acceptability, and, during this pandemic, gaps in public health monitoring and surveillance data [5], particularly from rural communities, have emerged, leading to concerns related to data reliability.

On a positive note, resolving this is not intractable. Heatmaps of retail and payroll activity are unsurprisingly similar to population density. This is where the innate intertwining of public health and economic well-being around the “location, location, location” axis can be synergistic. For instance, by adjusting the distribution of testing capacity to also account for population density, we could improve monitoring and response to blunt the speed and spread of the virus while also safeguarding both retail activity and economic nerve centers across the country.
